# Sarco-Osteoporosis: Prevalence and Association with Frailty in Chinese Community-Dwelling Older Adults

**DOI:** 10.1155/2015/482940

**Published:** 2015-07-27

**Authors:** Yan-Jiao Wang, Yi Wang, Jun-Kun Zhan, Zhi-Yong Tang, Jie-Yu He, Pan Tan, Hui-Qian Deng, Wu Huang, You-Shuo Liu

**Affiliations:** Geriatric Department, The Second Xiang-Ya Hospital, Institute of Aging and Geriatric, Central South University, No. 139 Middle Renmin Road, Changsha, Hunan 410011, China

## Abstract

The aim was to apply AWGS criteria to estimate the prevalence of sarco-osteoporosis and investigate its relationship with frailty, in a sample of 316 community-dwelling Chinese older people. Regression analysis was performed using frailty as the dependent variable. The results showed that the prevalence rate of sarco-osteoporosis was 10.4% in older men and 15.1% in older women. ≧80 years old (OR 4.8; 95% CI, 3.05–10.76; *P* = 0.027), women (OR 2.6; 95% CI, 1.18–2.76; *P* = 0.036), and higher level of comorbidity (OR 3.71; 95% CI, 1.61–10.43; *P* = 0.021) were independently associated with the likelihood of being sarco-osteoporosis. In the frail group, sarco-osteoporosis occurred in 26.3% of men, in 38.5% of women, and in lower proportion in the prefrail (13.6% of men; 16.2% of women) and nonfrail group (1.6% of men; 1.9% of women) (*P* < 0.05, resp.). Furthermore, the likelihood of being frail/prefrail was substantially higher in the presence of sarco-osteoporosis (OR 4.16; 95% CI, 2.17–17.65; *P* = 0.019 in men; and OR 4.67; 95% CI, 2.42–18.86; *P* = 0.007 in women). The results indicate that patients with sarco-osteoporosis are more likely to be ≧80 yrs with higher burden of comorbidities and to have frailty/prefrailty, especially for women.

## 1. Introduction

Population ageing is accelerating rapidly worldwide. Frailty in the elderly is a major public health problem. It is a state of increased vulnerability to poor resolution of homoeostasis after a minor stressor event, which increases the risk of adverse outcomes, including falls, delirium, disability, long-term care, and death [1, 2]. Between a quarter and half of people older than 85 years are estimated to be frail [3]. For older community residents, effective frailty prevention may potentially reduce serious frailty-related injuries. Reducing frailty risk in older individuals is, therefore, an important public health objective. So a clinical need exists to optimally identify those who will develop frailty.

Sarcopenia and osteoporosis are two distinct characteristics seen in older patients and are highly prevalent among elderly patients with frailty [4, 5]. Sarcopenia, the age-related loss of skeletal muscle mass, is characterized by the deterioration of muscle quantity and quality leading to a gradual slowing of movement, a decline in strength and power, increased risk of fall-related injury, and often, frailty [4]. Osteoporosis is a progressive bone disease that is characterized by a decrease in bone mass and density which can lead to an increased risk of fracture. In osteoporosis, the bone mineral density (BMD) is reduced, bone microarchitecture deteriorates, and the amount and variety of proteins in bone are altered. Osteoporosis is a common condition in elders and a powerful risk factor for adverse health outcomes such as fracture [5]. Intuitively, having a low muscle mass and strength with low bone mineral density (BMD) seems likely to lead to more physical functional limitations and frailty. However, we do not know the clinical characteristics of the individuals with both osteoporosis and sarcopenia, so-called “sarco-osteoporosis.” Despite sharing common risk factors and biological pathways, the relationship between frailty and sarco-osteoporosis is not clear. More research is needed to better understand sarco-osteoporosis. The objective of our present study was to investigate the prevalence of sarco-osteoporosis among community-dwelling Chinese elders and the relationship between sarco-osteoporosis and frailty.

## 2. Material and Methods

### 2.1. Participants

From August 2012 to August 2014, the patients who conducted comprehensive geriatric assessment (CGA) from community-dwelling Chinese elders (≧65 years) were recruited in Changsha city and its surrounding area of China. The enrollment work was done by a full-time nurse responsible for CGA. Individuals were originally excluded if unable to walk without the assistance of another person, or their renal function and liver function was abnormal, or their heart function classification was of grades III and IV according to New York Heart Association (NYHA) standard. Patients with severe parkinsonism were also excluded who had signs of postural instability. A total of 360 subjects were screened and 316 of them had sufficient data for analysis, and their characteristics are presented in Table 1. The study protocol was approved by the Second Xiangya Hospital of Central South University Ethics Committees in accordance with the Declaration of Helsinki and Good Clinical Practices Guidelines.

### 2.2. Assessment Methods

#### 2.2.1. Questionnaire about Health Status

Participants completed a questionnaire and were interviewed by a CGA nurse at the examination center and asked about health status, educational achievement, and smoking status. A selected medical history including a history of a physician diagnosis of diabetes mellitus, hypertension, coronary heart disease, dementia, parkinsonism, stroke, cancer, and chronic obstructive lung disease was obtained. Body weight and height measurements were used to calculate a standard body mass index (BMI).

#### 2.2.2. Frailty Status

Participants were classified as frail, prefrail, and nonfrail according to a validated screening tool based on the presence or absence of five measurable characteristics by Fried and colleagues [6]: weakness, low physical activity, slowed walking speed, exhaustion, and weight loss. (1) Weakness was defined as grip strength in the lowest quintile within groups defined by sex and BMI. Participants reported their level of daily leisure physical activity in the past year; (2) low physical activity was defined as either complete inactivity or performing low-intensity activities less than 1 h/wk; (3) slowed walking speed was defined as usual walking speed in the slowest quintile within groups defined by sex and height. Walking speed was measured on a 4 m course using photocell recordings at the course start and finish. The final measure averaged two walks; (4) exhaustion was indicated by a response of “occasionally” or “often/always” to the statement, “I felt that everything was an effort”; (5) weight loss was measured as self-reported unintentional weight loss more than 4.5 kg within the past year. Individuals with three or more of the five components were defined as frail, those with one or two components were defined as prefrail, and those with none were defined as nonfrail.

#### 2.2.3. Sarcopenia

Sarcopenia was defined as proposed by the Asian Working Group for Sarcopenia (AWGS) [7] with cutoff values for muscle mass measurements (7.0 kg/m^2^ for men and 5.7 kg/m^2^ for women by using bioimpedance analysis), handgrip strength (<26 kg for men and <18 kg for women), and usual gait speed (<0.8 m/s).

#### 2.2.4. Osteoporosis

Bone mineral density (BMD) of lumbar spine and femoral neck of all elderly adults were measured by DXA using QDR 4500A fan beam bone densitometer (Hologic Inc., Bedford, MA, USA), according to the manufacturer's recommended standard analysis procedures for the PA lumbar spine (vertebrae L2–L4) and hip femoral neck. A long-term (exceeding 15 years) coefficient of variation (CV) for the BMD was not greater than 0.40%. With reference to the World Health Organization (WHO) definition [5], the diagnosis of osteoporosis was established when a BMD of 2.5 SD was lower than the peak mean of the same gender (*T* ≤ −2.5), and secondary osteoporosis was excluded.

#### 2.2.5. Covariates

Covariates were selected if they were considered to be related to frailty status, low bone mineral density, or sarcopenia. The covariates used were age, education (less than 9 years and more than 9 years), drinking and smoking (current drinking or current smoking or if stopped less than 4 years prior to the interview), supplemental Vitamin D use or supplemental calcium use (less than twice every week and more than twice every week), physical activity (less than 30 minutes per day and more than 30 minutes per day), and the number of chronic diseases including diabetes mellitus, hypertension, coronary heart disease, dementia, parkinsonism, stroke, cancer, and chronic obstructive lung disease (less than 3 and more 3).

### 2.3. Statistical Analysis

Descriptive statistics were reported as mean ± standard deviation. Data were analyzed including distribution of means and proportions of variables of interest across sex, musculoskeletal diseases, or frailty categories were compared using the student *t*-test and Chi-square and trend tests. Logistic regression using sarco-osteoporosis as the dependent variable for characteristics of the subjects, or using frailty/prefrailty (or nonfrailty) as the dependent variable for groups of musculoskeletal diseases classification, and adjusted for covariates was performed. “No sarcopenia and no osteoporosis” was set as the reference group. Results were presented as odds ratio (OR) with 95% confidence intervals (CIs). All analyses were performed using the SPSS 19.0 package (SPSS, Chicago, IL). *P* < 0.05 was considered to be statistically significant.

## 3. Results

This study analyzed 316 elders who had sufficient data for analysis. The percentage of women was 48% (*n* = 152). Characteristics of the subjects by sex are shown in Table 1. The mean age was 75.6 ± 4.8 years for men and 74.9 ± 5.2 years for women, and the average BMI value was 23.1 kg/m^2^ and 23.6 kg/m^2^ in men and women, respectively, with no significant difference between the two groups. In the total body bioimpedance analysis, women presented a higher percentage of total fat mass as compared to men. Men had significantly higher values than did women for all BMD measurements, grip strength, and walking speed. The proportions of current drinker or current smoker and chronic obstructive lung disease were higher in men than in women.

However, the percentage of physically active individuals was lower in men than in women. There was no significant difference in education, vitamin D and calcium supplementation, proportions of diabetes, hypertension coronary heart disease, dementia, parkinsonism, stroke, and cancer among the two groups.

Subject characteristics for sarco-osteoporosis are shown in Tables 2 and 3. 26.2% of men (*n* = 43) and 33.6% of women (*n* = 51) were classified as having sarcopenia (having osteoporosis or no osteoporosis); 29.3% (*n* = 48) of men and 46.1% (*n* = 70) of women were classified as having osteoporosis (having sarcopenia or no sarcopenia). Sarco-osteoporosis was prevalent in 10.4% of men (*n* = 17) and 15.1% of women (*n* = 23) among the study population. The mean age of men or women in the sarco-osteoporosis group was significantly higher than the mean age of those in the other groups. The percentage of drinkers, smokers, or parkinsonism was significantly higher in the sarco-osteoporosis group than in the other groups among men.

In a logistic regression model, ≧80 years old or women had an increased likelihood of sarco-osteoporosis compared to <80 years old or men (OR 4.8; 95% CI, 3.05–10.76; *P* = 0.027; OR: 2.6; 95% CI, 1.18–2.76; *P* = 0.036, resp.). Moreover, higher level of comorbidity (the number of chronic diseases was more than 3) was associated with sarco-osteoporosis (OR 3.71; 95% CI, 1.61–10.43; *P* = 0.021).

Subject characteristics for frailty/prefrailty stratified by sex are shown in Tables 4 and 5. Frailty status was detected in 11.6% (*n* = 19) of the elderly men, prefrailty in 49.4% (*n* = 81), and nonfrailty in 39.0% (*n* = 64) of the elderly men and frailty in 17.1% (*n* = 26) of the elderly women, prefrailty in 48.7% (*n* = 74), and nonfrailty in 34.2% (*n* = 52) of the elderly women. In the frail group, sarco-osteoporosis occurred in 26.3% of men (*n* = 5), 38.5% of women (*n* = 10), and in lower proportion in the prefrail (13.6% of men, *n* = 11; 16.2% of women, *n* = 12) and the nonfrailty groups (1.6% of men, *n* = 1; 1.9% of women, *n* = 1) (Tables 4 and 5). The mean age of men or women in the frailty/prefrailty group was significantly higher than that of those in the nonfrailty group.

The associations between sarco-osteoporosis and frailty/prefrailty in men and women, as assessed by logistic regression analysis, are shown in Tables 6 and 7, respectively. After adjusting for subjects aged 80 years or more, drinking and smoking, education, body mass index and percentage of fat in the whole body scan, chronic medical history, sarcopenia (OR 3.11; 95% CI, 1.65–6.63; *P* = 0.018 in men; OR 3.38; 95% CI, 1.41–7.62; *P* = 0.025 in women), and osteoporosis (OR 2.07; 95% CI, 1.09–13.12; *P* = 0.037 in men; OR 2.41; 95% CI, 1.26–14.15; *P* = 0.024 in women) were independently associated with frailty. Furthermore, the likelihood of being frail was substantially higher in the presence of sarco-osteoporosis (OR 4.16; 95% CI, 2.17–17.65; *P* = 0.019 in men; and OR 4.67; 95% CI, 2.42–18.86; *P* = 0.007 in women.) (Tables 6 and 7).

## 4. Discussion

This cross-sectional study examined the association between sarco-osteoporosis (the individuals with both sarcopenia and osteoporosis, as diagnosed by AWGS/WHO criteria) and frailty in 316 community-dwelling elderly Chinese men and women. We found that, in the frail group, sarco-osteoporosis occurred in 26.3% of men and 38.5% of women, but in lower proportion in the prefrail group (13.6% of men and 16.2% of women) or in the nonfrailty group (1.6% of men and 1.9% of women). In other words, the percentages of sarco-osteoporosis were higher in the frailty/prefrailty groups than in the nonfrailty group in both men and women. Furthermore, the likelihood of being frail/prefrail was substantially higher in the presence of sarco-osteoporosis (OR 4.16; 95% CI, 2.17–17.65; *P* = 0.019 in men; and OR 4.67; 95% CI, 2.42–18.86; *P* = 0.007 in women) than in the presence of sarcopenia or osteoporosis alone (OR 3.11; 95% CI, 1.65–6.63; OR 2.07, 95% CI, 1.09–13.12 in men and OR 3.38; 95% CI, 1.41–7.62; *P* = 0.025; OR 2.41; 95% CI, 1.26–14.15; *P* = 0.024 in women, resp.) compared with neither sarcopenia nor osteoporosis.

Many elderly people have multiorgan problems. Frailty is a practical, unifying notion in the care of elderly adults that directs attention away from single-system illness towards a more holistic viewpoint of the patient. Reduction of the occurrence or severity of frailty is likely to have large benefits for individuals, their families, and society. Some clinical trials have confirmed that complex interventions including exercise, nutrient supplement, and pharmacological agents can increase the likelihood of continuing to live at home, mainly through a reduced need for care-home admission and fewer falls [8, 9]. To identify the subjects with high risk factors for frailty should be an essential part of further complex intervention. The immune system, skeletal muscle, brain, and endocrine system are intrinsically interrelated and are the organ systems that are best studied in the development of frailty [6]. Notably, frailty has also been associated with loss of physiological reserve in the cardiovascular [10], renal [11], respiratory [12], haemopoietic, and clotting systems [13, 14], and nutritional status can also be a mediating factor [1, 15–17]. The results from our study implied that sarcopenia and osteoporosis were predictors of frailty. Importantly, the results suggested that the joint predictive value of sarcopenia and osteoporosis was stronger than that of sarcopenia or osteoporosis alone. This finding supports the idea that, when physiological decline reaches an aggregate crucial level, frailty becomes evident [18].

The results from the Women's Health and Aging Study (WHAS) II showed almost sixteen percent had sarcopenia concomitant to severe osteopenia/osteoporosis in community-dwelling older women according to Baumgartner/WHO criteria [19]. Our present study for the first time showed the epidemiology of sarco-osteoporosis and its associative clinical characteristics in community-dwelling elderly Chinese men and women. Sarco-osteoporosis was prevalent in 10.4% of men and 15.1% of women among the study population. Sarco-osteoporosis prevalence was lower than that of isolated sarcopenia (15.9% of men and 18.4% of women) or isolated osteoporosis alone (18.9% of men and 30.9% of women). We also found that ≧80 years old or women had an increased likelihood of sarco-osteoporosis compared to <80 years old or men (OR 4.8, 95% CI: 3.05–10.76, *P* = 0.027; OR 2.6, 95% CI: 1.18–2.76, *P* = 0.036, resp.). Moreover, higher level of comorbidity (the number of chronic diseases was more than 3) was associated with sarco-osteoporosis (OR 3.71, 95% CI: 1.61–10.43, *P* = 0.021).

Muscle/bone relationships have recently been noted as a new research field related to the interactions among several organ systems. Muscle/bone relationships include two factors: local control of muscle to bone and systemic humoral interactions between muscle and bone. Genetic, endocrine, and mechanical factors affect both muscle and bone simultaneously. Further progress in understanding the common genetic etiology of osteoporosis and sarcopenia will provide valuable insight into important biological underpinnings for both conditions and may translate into new approaches to reduce the burdens of both conditions through improved diagnosis, prevention, and early targeted treatment. [20]. Osteoporosis and sarcopenia may be affected by genetic polymorphisms of several genes, such as androgen receptor, estrogen receptor, catechol-O-methyltransferase, IGF-I, Vitamin D receptor, and low-density-lipoprotein receptor-related protein 5 [20]. Vitamin D [21], the growth hormone/insulin-like growth factor *I* axis [22], and testosterone [23] are physiologically and pathologically important as endocrine factors. Mechanical stress changes, such as immobilization and lack of gravity, greatly influence both muscle and bone [24]. These findings suggest the presence of interactions between muscle and bone, which might be very important for understanding the physiology and pathophysiology of sarco-osteoporosis. The loss of muscle strength and mass during the aging process causes structural changes in the microarchitecture of the bones and decreases mineral density, resulting in bone quality decline. These factors of skeletal muscle and bone activate a vicious cycle leading to accelerated frailty and ultimately to physical disability.

There are shared factors between sarcopenia and frailty such as slow walking speed and grip strength. If sarcopenia is integrated into the diagnosis of frailty, it would be positively identified and graded for severity. And it would help essential research to gain a deeper insight into the complex mechanisms of frailty and aid the development and evaluation of interventions to improve outcomes.

This study had several advantages over previous studies. First, the subjects were recruited from a community-based elderly population, represented a single Chinese older adults. Second, previous studies used EWGS criteria for the definition of sarcopenia to obtain a sufficient number of subjects within the group for statistical analysis [25]. In contrast, we used the criterion of AWGS for defining sarcopenia, which is known to be more suitable for Chinese [7]. But the present study has three limitations. First, the sample size of the subgroups in the analysis is relatively small and provides limited statistical power, and further investigation of the joint effects of sarcopenia and osteoporosis on frailty is needed. Second, the individuals were originally excluded if unable to walk without the assistance of another person, or their renal function and liver function is abnormal, or their heart function classification is of grades III and IV according to NYHA standard; this may have biased our results towards an underestimation of the risk of frailty associated with sarcoosteopenia. Third, DXA scan presents some limitations in BMD evaluation in elderly people, like aortic calcifications and spine osteoarthritis that may produce an increase, up to 10%, in BMD of the lumbar spine [26], and this could underestimate the real prevalence of sarco-osteoporosis in this population and, consequently, the association with frailty status. Therefore, findings from this study should be interpreted in the context of the complexity of skeletal muscle and bone as well as multifactorial nature of the frailty syndrome. Despite these limitations, our findings are helpful for us to better understand sarco-osteoporosis and provide a basis for making an optimal prediction about frailty among community-dwelling Chinese older people.

## 5. Conclusion

In conclusion, sarco-osteoporosis defined by AWGS/WHO criteria is present in 10.4% of men and 15.1% of women aged over 65 years, and its prevalence rate is higher in community-dwelling Chinese people aged 80 and over. The joint effect of sarcopenia and osteoporosis may be tightly linked to the risk of frailty. Assessment of both bone and muscle mass/function in older adults could potentially enhance frailty risk prediction. Further prospective study is needed to clarify the roles of sarco-osteoporosis in the occurrence of frailty and frailty-related health outcomes. Although no causal attribution is possible in this analysis, sarco-osteoporosis may explain some of the increases in frailty risk currently related to “age.” Therefore, it is appropriate to consider sarcopenia together with osteoporosis in the elderly population.

## Figures and Tables

**Table 1:** Characteristics of the subjects by sex.

	Men *n* = 164	Women *n* = 152	*P* value
Age (years)	75.6 ± 4.8	76.9 ± 5.2	Matched
BMI (kg/m^2^)	23.1 ± 2.5	23.6 ± 3.4	0.357
AMI (kg/m^2^)	7.7 ± 0.6	6.2 ± 0.7	0.031
Body fat (%)	23.3 ± 4.9	29.9 ± 5.7	0.035
Grip strength (kg)	34.9 ± 5.2	23.6 ± 3.8	0.017
Usual walking speed (m/s)	1.56 ± 0.3	1.41 ± 0.3	0.012
BMD (g/cm^2^)			
Lumbar spine	0.81 ± 0.07	0.76 ± 0.07	0.037
Femoral neck	0.63 ± 0.06	0.58 ± 0.07	0.013
Education (>9 years)	36 (22.0)	32 (21.1)	0.583
Supplemental Vitamin D use	38 (23.1)	41 (27.0)	0.089
Supplemental calcium use	43 (26.2)	44 (28.9)	0.237
Lifestyle related habits			
Current drinker	25 (15.2)	7 (4.6)	0.027
Current smoker	32 (19.5)	5 (3.3)	0.019
Physically active	37 (22.6)	41 (27.0)	0.174
Chronic medical history			
Diabetes	38 (23.1)	32 (21.1)	0.318
Hypertension	53 (32.3)	49 (32.2)	0.671
Coronary heart disease	31 (18.9)	27 (17.8)	0.484
Dementia	3 (1.8)	3 (2.0)	0.375
Parkinsonism	6 (3.7)	5 (3.3)	0.278
Stroke	4 (2.4)	3 (3.3)	0.069
Cancer	2 (1.2)	2 (1.3)	0.427
Chronic obstructive lung disease	19 (11.6)	12 (7.9)	0.038
Musculoskeletal diseases classification			
Sarcopenia and no osteoporosis	26 (15.9)	28 (18.4)	0.387
Osteoporosis and no sarcopenia	31 (18.9)	47 (30.9)	0.016
Sarco-osteoporosis	17 (10.4)	23 (15.1)	0.024
No sarcopenia and no osteoporosis	90 (54.8)	54 (35.6)	0.018

*Notes.*Values are mean ± standard deviation (SD) and number (percentage). BMI: body mass index; AMI: appendicular muscle mass index; BMD: bone mineral density.

**Table 2:** Subject characteristics of men by musculoskeletal diseases classification.

Men *n* = 164	No sarcopenia and no osteoporosis *n* = 90	Sarcopenia and no osteoporosis *n* = 26	Osteoporosis and no sarcopenia *n* = 31	Sarco-osteoporosis *n* = 17	*P* value
Age (years)	71.1 ± 3.4	78.6 ± 3.8	74.8 ± 4.3	83.1 ± 2.9	0.012
BMI (kg/m^2^)	24.7 ± 2.4	22.6 ± 2.1	24.5 ± 3.3	21.7 ± 2.8	0.273
AMI (kg/m^2^)	8.6 ± 1.4	6.7 ± 0.3	7.1 ± 1.1	6.1 ± 0.4	0.013
Body fat (%)	21.6 ± 2.7	28.9 ± 2.2	23.7 ± 2.6	30.7 ± 2.4	0.011
Grip strength (kg)	35.9 ± 5.2	23.9 ± 2.3	30.6 ± 3.1	21.1 ± 2.0	0.001
Walking speed (m/s)	1.61 ± 0.22	0.72 ± 0.13	1.56 ± 0.17	0.63 ± 0.19	0.016
BMD (g/cm^2^)					
Lumbar spine	0.84 ± 0.06	0.82 ± 0.06	0.73 ± 0.09	0.72 ± 0.11	0.041
Femoral neck	0.71 ± 0.07	0.69 ± 0.05	0.62 ± 0.08	0.60 ± 0.13	0.028
Education (>9 years)	16 (17.8%)	7 (27.0%)	8 (25.9%)	5 (29.4%)	0.156
Supplemental Vitamin D use	15 (16.7%)	10 (38.5%)	9 (29.0%)	4 (23.6%)	0.231
Supplemental calcium use	17 (18.9%)	12 (46.2%)	10 (32.3%)	4 (23.6%)	0.369
Lifestyle related habits					
Drinker	7 (7.8%)	4 (15.4%)	6 (19.4%)	8 (47.1%)	0.023
Smoker	10 (11.1%)	6 (23.1%)	9 (29.0%)	7 (41.2%)	0.027
Physically active	18 (20.0%)	5 (19.2%)	8 (25.8%)	6 (15.3%)	0.159
Chronic medical history					
Diabetes	13 (14.4%)	9 (34.6%)	10 (32.3%)	6 (35.3%)	0.057
Hypertension	16 (17.8%)	14 (53.9%)	13 (42.0%)	10 (58.8%)	0.063
CHD	14 (15.6%)	6 (23.1%)	8 (25.8%)	3 (17.6%)	0.326
Dementia	0	1 (3.8%)	1 (3.2%)	1 (5.9%)	0.417
Parkinsonism	1 (1.1%)	2 (7.7%)	1 (3.2%)	2 (11.8%)	0.046
Stroke	1 (1.1%)	1 (3.8%)	2 (6.5%)	0	0.132
Cancer	1 (1.1%)	0	1 (3.2%)	0	0.413
COPD	4 (4.4%)	7 (27.0%)	5 (16.1%)	3 (17.6%)	0.247

*Notes.* Values are mean ± standard deviation (SD) and number (percentage). BMI: body mass index; AMI: appendicular muscle mass index; BMD: bone mineral density; CHD: coronary heart disease; COPD: chronic obstructive lung disease.

**Table 3:** Subject characteristics of women by musculoskeletal diseases classification.

Women *n* = 152	No sarcopenia and no osteoporosis *n* = 54	Sarcopenia and no osteoporosis *n* = 28	Osteoporosis and no sarcopenia *n* = 47	Sarco-osteoporosis *n* = 23	*P* value
Age (years)	73.2 ± 2.8	77.2 ± 3.1	73.8 ± 3.7	82.5 ± 2.6	0.017
BMI (kg/m^2^)	25.4 ± 2.1	21.1 ± 2.6	23.6 ± 3.8	22.3 ± 3.4	0.338
AMI (kg/m^2^)	7.2 ± 0.7	5.2 ± 0.3	7.0 ± 0.4	5.1 ± 1.2	0.025
Body fat (%)	28.1 ± 3.7	30.7 ± 2.1	27.9 ± 3.2	32.9 ± 2.3	0.014
Grip strength (kg)	25.9 ± 2.2	13.9 ± 1.6	22.6 ± 1.3	13.1 ± 0.9	0.015
Walking speed (m/s)	1.64 ± 0.17	0.69 ± 0.12	1.46 ± 0.11	0.62 ± 0.09	0.008
BMD (g/cm^2^)					
Lumbar spine	0.79 ± 0.07	0.72 ± 0.05	0.63 ± 0.03	0.62 ± 0.05	0.021
Femoral neck	0.67 ± 0.04	0.65 ± 0.03	0.59 ± 0.08	0.60 ± 0.05	0.016
Education (>9 years)	13 (24.1%)	6 (21.4%)	10 (21.3%)	3 (13.0%)	0.156
Supplemental Vitamin D use	16 (29.6%)	14 (50.0%)	7 (14.9%)	4 (17.4%)	0.231
Supplemental calcium use	17 (31.5%)	13 (46.4%)	10 (21.3%)	4 (17.4%)	0.369
Lifestyle related habits					
Drinker	2 (3.7%)	2 (7.1%)	2 (4.3%)	1 (4.3%)	0.023
Smoker	2 (3.7%)	1 (3.6%)	2 (4.3%)	0	0.027
Physically active	17 (31.5%)	8 (28.6%)	10 (21.3%)	6 (26.1%)	0.159
Chronic medical history					
Diabetes	10 (18.5%)	7 (25.0%)	9 (19.1%)	6 (26.1%)	0.137
Hypertension	19 (35.2%)	7 (25.0%)	17 (36.2%)	6 (26.1%)	0.308
CHD	10 (18.5%)	6 (21.4%)	8 (17.0%)	3 (13.0%)	0.289
Dementia	1 (1.9%)	1 (3.6%)	0	1 (4.3%)	0.165
Parkinsonism	2 (3.7%)	1 (3.6%)	1 (2.1%)	1 (4.3%)	0.148
Stroke	1 (1.9%)	1 (3.6%)	0	1 (4.3%)	0.189
Cancer	1 (1.9%)	1 (3.6%)	0	0	0.275
COPD	5 (9.3%)	3 (10.7%)	3 (6.4%)	1 (4.3%)	0.158

*Notes*. Values are mean ± standard deviation (SD) and number (percentage). BMI: body mass index; AMI: appendicular muscle mass index; BMD: bone mineral density; CHD: coronary heart disease; COPD: chronic obstructive lung disease.

**Table 4:** Characteristics of men by presence of frailty/prefrailty.

Men *n* = 164	Frailty *n* = 19	Prefrailty *n* = 81	Nonfrailty *n* = 64	*P* value
Age (years)	83.5 ± 2.9	76.1 ± 3.5	72.1 ± 3.3	0.017
AMI (kg/m^2^)	5.4 ± 1.3	6.8 ± 1.6	7.9 ± 1.4	0.014
Body fat (%)	28.6 ± 2.2	27.3 ± 2.0	24.1 ± 2.1	0.036
Grip strength (kg)	22.6 ± 1.9	28.1 ± 2.6	33.6 ± 2.0	0.009
Usual walking speed (m/s)	0.64 ± 0.1	0.83 ± 0.3	1.52 ± 0.4	0.003
BMD (g/cm^2^)				
Lumbar spine	0.75 ± 0.09	0.79 ± 0.11	0.82 ± 0.8	0.032
Femoral neck	0.63 ± 0.10	0.67 ± 0.13	0.72 ± 0.11	0.037
Musculoskeletal diseases classification				
Sarcopenia and no osteoporosis (*n* = 26)	4 (21.1%)	20 (24.7%)	2 (3.1%)	0.004
Osteoporosis and no sarcopenia (*n* = 31)	5 (26.3%)	22 (27.2%)	4 (6.3%)	0.015
Sarco-osteoporosis (*n* = 17)	5 (26.3%)	11 (13.6%)	1 (1.6%)	0.003
No sarcopenia and no osteoporosis (*n* = 90)	5 (26.3%)	28 (34.6%)	57 (89.1%)	0.012

*Notes.* Values are mean ± standard deviation (SD) and number (percentage). AMI: appendicular muscle mass index; BMD: bone mineral density.

**Table 5:** Characteristics of women by presence of frailty/prefrailty.

Women *n* = 152	Frailty *n* = 26	Prefrailty *n* = 74	Nonfrailty *n* = 52	*P* value
Age (years)	84.7 ± 2.6	77.1 ± 3.1	74.1 ± 3.9	0.023
AMI (kg/m^2^)	5.0 ± 1.6	6.3 ± 1.9	7.2 ± 1.8	0.019
Body fat (%)	33.5 ± 2.7	28.6 ± 2.9	27.4 ± 2.5	0.026
Grip strength (kg)	13.6 ± 1.9	17.1 ± 2.6	24.6 ± 2.0	0.006
Usual walking speed (m/s)	0.65 ± 0.13	0.86 ± 0.09	1.62 ± 0.19	0.003
BMD (g/cm^2^)				
Lumbar spine	0.64 ± 0.11	0.69 ± 0.16	0.72 ± 0.07	0.038
Femoral neck	0.61 ± 0.16	0.65 ± 0.08	0.71 ± 0.14	0.031
Musculoskeletal diseases classification				
Sarcopenia and no osteoporosis (*n* = 28)	7 (26.9%)	19 (25.7%)	2 (3.8%)	0.007
Osteoporosis and no sarcopenia (*n* = 47)	8 (30.8%)	26 (35.1%)	13 (25.0%)	0.028
Sarco-osteoporosis (*n* = 23)	10 (38.5%)	12 (16.2%)	1 (1.9%)	0.004
No sarcopenia and no osteoporosis (*n* = 54)	1 (3.8%)	17 (23.0%)	36 (69.2%)	0.013

*Notes*. Values are mean ± standard deviation (SD) and number (percentage). AMI: appendicular muscle mass index; BMD: bone mineral density.

**Table 6:** Association between sarco-osteoporosis, sarcopenia, or osteoporosis and frailty/prefrailty in men.

Men	Frailty or prefrailty/nonfrailty		
	Odds ratio	95% Cl	*P *value
No sarcopenia and no osteoporosis	1		
Sarcopenia	3.11	1.65–6.63	0.018
Osteoporosis	2.07	1.09–13.12	0.037
Sarco-osteoporosis	4.16	2.17–17.65	0.019

**Table 7:** Association between sarco-osteoporosis, sarcopenia, or osteoporosis and frailty/prefrailty in women.

Women	Frailty or prefrailty/nonfrailty		
	Odds ratio	95% Cl	*P *value
No sarcopenia and no osteoporosis	1		
Sarcopenia	3.38	1.41–7.62	0.025
Osteoporosis	2.41	1.26–14.15	0.024
Sarco-osteoporosis	4.67	2.42–18.86	0.007
